# Low rates of antibiotic use among ambulatory patients with coronavirus disease 2019 (COVID-19)

**DOI:** 10.1017/ash.2022.17

**Published:** 2022-04-11

**Authors:** Alison G.C. Smith, Eli Wilber, Paulina A. Rebolledo, Joseph Sharp, Sheetal Kandiah, Daniel S. Graciaa, Russell R. Kempker

**Affiliations:** 1 Emory University School of Medicine, Atlanta, Georgia; 2 Division of Infectious Diseases, Department of Medicine, Emory University School of Medicine, Atlanta, Georgia; 3 Hubert Department of Global Health, Rollins School of Public Health, Emory University, Atlanta, Georgia; 4 Division of General Medicine and Geriatrics, Department of Medicine, Emory University School of Medicine, Atlanta, Georgia

## Abstract

We assessed the prevalence of antibiotic prescriptions among ambulatory patients tested for coronavirus disease 2019 (COVID-19) in a large public US healthcare system and found a low overall rate of antibiotic prescriptions (6.7%). Only 3.8% of positive severe acute respiratory coronavirus virus 2 (SARS-CoV-2) tests were associated with an antibiotic prescription within 7 days.

The coronavirus disease 2019 (COVID-19) pandemic has presented a major challenge to antimicrobial stewardship efforts worldwide. Multiple studies have illustrated the widespread use of broad-spectrum antibiotics for patients with COVID-19 in the inpatient setting despite low rates of confirmed bacterial coinfection,^1^ but far less is known about antibiotic utilization for nonhospitalized patients with suspected or confirmed COVID-19. Observational studies in the United States and the United Kingdom have reported declining outpatient antibiotic prescriptions during the COVID-19 pandemic.^2,3^ However, data from the United Kingdom suggest high rates of 37% for outpatient antibiotic use during COVID-19 illness in patients who eventually required hospitalization in the context of national guidelines recommending doxycycline or amoxicillin for high-risk COVID-19 pneumonia.^4,5^

In the United States, prior data on outpatient antibiotic stewardship during the COVID-19 pandemic is extremely limited. In a single-center review of 346 patients that examined antibiotic use across the healthcare continuum in March and April 2020, only 3% of patients diagnosed with COVID-19 during an ambulatory encounter were prescribed antibiotics for respiratory illness within 14 days of testing.^6^ Additional data are necessary to determine whether antibiotics are being prescribed appropriately for ambulatory COVID-19. We sought to help fill this knowledge gap by determining outpatient antibiotic prescription rates among patients tested for severe acute respiratory coronavirus virus 2 (SARS-CoV-2) at a large safety-net healthcare system in the southeastern United States.

## Methods

Our study population included all ambulatory patients tested for SARS-CoV-2 within the Grady Health System (GHS) from April 1, 2020, through June 30, 2021. GHS is a large publicly funded safety-net healthcare system in Atlanta, Georgia, that includes Grady Memorial Hospital and its primary care clinic, 6 neighborhood health centers, a large HIV clinic, and multiple medical and surgical specialty clinics. In April 2020, outpatient testing for SARS-CoV-2 was implemented utilizing polymerase chain reaction (PCR) assay on nasopharyngeal specimens at the Grady Microbiology Laboratory using the Abbott Laboratories *m*2000 RealTIME (Lake Bluff, IL) system or the Abbot Laboratories Alinity SARS-CoV-2 RT-PCR system. Data extraction from the Grady EPIC electronic medical record system was performed by the Grady information technology team. The database comprised all ambulatory patients with a SARS-CoV-2 test including clinic visits, telehealth encounters, and testing-only encounters. Testing conducted through GHS employee health services was excluded from analysis. The following data were extracted: demographics, SARS-CoV-2 test dates and results, antibiotic prescriptions, associated outpatient visit records, and records of inpatient admissions in the 30 days following SARS-CoV-2 testing. Antibiotic prescriptions within 7 days of a SARS-COV-2 test (before or after) were included. The National Healthcare Safety Network Antimicrobial Use Protocol was used to define antimicrobial inclusion,^7^ and antifungal agents and antibiotics prescribed for HIV infection-related opportunistic infection prophylaxis were additionally excluded. For all patients with a positive SARS-CoV-2 test who received antibiotics, 2 physicians conducted chart review and independently determined appropriateness of antibiotic prescriptions. A third board-certified infectious disease physician made a final determination in the case of disagreement.

## Results

During the 15-month study period, 21,566 SARS-CoV-2 PCR tests were performed among 15,113 unique persons, with 1,437 positive test results (6.7%). Among all testing encounters 34.2% were associated with an outpatient clinic visit within 7 days. In total, 1,381 antibiotic prescriptions were ordered within 7 days of SARS-CoV-2 testing (64 per 1,000 patient encounters). Antibiotics were more commonly prescribed in association with a negative (6.6%) versus positive (3.8%) SARS-CoV-2 test (*P* < .001). Patients who were white and those with private insurance were more likely to receive antibiotics within 7 days of their SARS-CoV-2 test compared with patients who were black or African American, and patients with Medicaid or Medicare, respectively (Table 1).

**Table 1.** Characteristics of Patients Undergoing Outpatient COVID-19 by Receipt of Antibiotics Among All SARS-CoV-2 Testing Encounters

Variable	Overall, (N=21,566), No. (%)^a^	Did not receive antibiotics, (N=20,185), No. (%)^a^	Received antibiotics, (N=1,381), No. (%)^a^	*P* Value^b^
No. of unique patients	15,113	13,755	1,358	
**SARS-CoV-2 test result**				<.001
Negative	20,129 (93)	18,803 (93.4)	1,326 (6.6)	
Positive	1,437 (6.7)	1,382 (96.2)	55 (3.8)	
Age, mean y (SD)	53 (14)	54 (14)	48 (16)	<.001
**Race**				<.001
White	1,637 (7.6)	1,478 (90.3)	159 (9.7)	
Black or African American	17,749 (82)	16,649 (93.8)	1,100 (6.2)*	
Hispanic	1,507 (7.0)	1,424 (94.5)	83 (5.5)	
Other	508 (2.4)	478 (94.1)	30 (5.9)	
Missing or refused	165 (0.8)	156 (94.5)	9 (5)	
**Language**				.013
English	19,828 (92)	18,532 (93.5)	1,296 (6.5)	
Spanish	1,250 (5.8)	1,181 (94.5)	69 (5.5)	
All others	439 (2.0)	424 (96.6)	15 (3.4)	
Missing or unknown	49 (0.2)	48 (98.0)	1 (2.0)	
HIV status^c^	2,495 (12)	2,351 (94.2)	144 (5.8)	.284
**Characteristics of prescription**				
Time between SARS-CoV-2 test and receipt of any prescription, mean d (absolute value) (SD)	2.78 (2.34)	2.85 (2.50)	2.66 (2.00)	.7467
**Characteristics of clinic visit**				
Had an outpatient clinic visit within 7 d of SARS-CoV-2 test	7,378 (34.2)	6,657 (90.2)	721 (9.8)	<.001
Time between test and visit, (absolute value), mean d (SD)	2.20 (2.44)	2.22 (2.45)	1.98 (2.39)	.020
**Payment method^d^**				<.001
Private insurance	2,353 (15)	2,181 (92.7)	172 (7.3)	
Medicaid and Medicare	7,494 (48)	7,076 (94.4)	418 (5.6)**	
Self-pay	5,876 (37)	5,493 (93.5)	383 (6.5)	

Note. HIV, human immunodeficiency virus.

b Statistical tests performed as indicated: 2-sample test for equality of proportions, χ^2^ test of independence, Fisher exact test, and Wilcoxon rank-sum test.

c Participants were considered HIV-positive if they had any CD4 testing performed at the GHS outpatient HIV clinic or if they had a CD4 count < 200 at any other GHS clinic site.

d Payment data available only for COVID-19 tests with associated outpatient visits.

* *P* < .001. Comparison of the proportion of white vs black or African-American patients who received antibiotics: 2-sample test for equality of proportions with continuity correction.

** *P* = .025. Comparison of the proportion of patients with private insurance vs self-paying patients who received antibiotics: 2-sample test for equality of proportions with continuity correction.

Among the 55 persons with COVID-19 and an associated antibiotic prescription, 21 (38.2%) had no appropriate indication for an antibiotic, and 12 (63.2%) of these inappropriate prescriptions were for azithromycin (Table 2). Telehealth visits were associated with a higher proportion of inappropriate antibiotic prescriptions than in-person visits (76.9% vs 28.2%; *P* = .006) (Table 2). The most common antibiotics prescribed among outpatients with a positive SARS-CoV-2 test were macrolides and penicillins (Supplementary Table 1 online). Inpatient admission rates at 7 and 30 days for patients who tested positive for SARS-COV-2 were similar among those who did and did not receive antibiotics (Supplementary Table 2 online).

**Table 2.** Characteristics of Antibiotic Prescriptions for Patients Who Received Antibiotics Within 7 Days of a Positive Outpatient SARS-CoV-2 Test Result

Variable	Overall (n=55), No (%)	Appropriate Antibiotic Prescription (n=34), No. (%)^a^	Inappropriate Antibiotic Prescription (n=21), No. (%)^a^
**Type of antibiotic prescribed**			
Azithromycin	19 (34.5)	7 (36.8)	12 (63.2)
Amoxicillin/clavulanate	7 (12.7)	5 (71.4)	2 (28.6)
All others	29 (52.7)	22 (75.9)	7 (24.1)
**Type of visit**			
In-person visit	39 (70.9)	28 (71.8)	11 (28.2)
Telehealth visit	13 (23.6)	3 (23.1)	10 (76.9)^b^
No visit documented	3 (5.5)	3 (100)	0 (0)
**Indication for SARS-CoV testing**			
Exposed	1 (1.8)	1 (100)	0 (0)
Symptomatic	33 (60.0)	16 (48.5)	17 (51.5)
Follow-up prior positive	5 (9.1)	2 (40.0)	3 (60.0)
Screening (includes preoperative)	15 (27.3)	15 (100)	0 (0)
Not clearly documented	1 (1.8)	0 (0)	1 (100)
**Indication for prescription**			
COVID-19–like illness^c^	4 (7.3)	0 (0)	4 (100)
Pneumonia	11 (20.0)	5 (45.5)	6 (54.5)
COVID-19 and concern for pneumonia	2 (3.6)	0 (0)	2 (100)
Other respiratory concern^d^	8 (14.5)	5 (62.5)	3 (37.5)
Nonrespiratory	30 (54.5)	24 (80.0)	6 (20.0)
**Febrile (>38.1°C)**			
Yes	3 (5.5)	1 (33.3)	2 (66.7)
No	27 (49.1)	20 (74.1)	7 (25.9)
Not documented	25 (45.5)	13 (52.0)	12 (48.0)
**When and where were antibiotics prescribed in relation to visit?**			
Before outpatient visit	6 (10.9)	4 (66.7)	2 (33.3)
After outpatient visit^e^	10 (18.2)	6 (60.0)	4 (04.0)
Same day as outpatient visit	32 (58.2)	19 (59.4)	13 (40.6)
In the emergency department	4 (7.3)	2 (50.0)	2 (50.0)
No visit documented	3 (5.5)	3 (100)	0 (0)

Note. COPD, chronic obstructive pulmonary disease.

a Units unless otherwise specified.

a Appropriateness of antibiotic prescription.

b Comparison of the proportion of inappropriate antibiotic prescriptions associated with telehealth visits vs. associated with in-person visits: 2-sample test for equality of proportions with continuity correction, *P* = .006.

c COVID-19–like illness was defined as documented concern for COVID-19 pneumonia or the presence of ≥2 of fever, myalgias and cough in absence of other known infectious diagnosis.

d Includes acute bacterial sinusitis, COPD exacerbation, “bronchitis,” and pharyngitis.

e “After” includes antibiotics prescribed after the initial clinic visit at a follow-up telehealth appointment.

## Discussion

Our findings demonstrate an overall low rate of antibiotic prescriptions among individuals presenting for outpatient SARS-COV-2 testing, with a significantly lower rate of antibiotic prescriptions among individuals diagnosed with COVID-19 (3.8% for SARS-CoV-2–positive vs 6.6% for SARS-CoV-2–negative). This finding differs from high rates of antibiotic use reported in patients hospitalized with COVID-19,^1^ but it aligns with recent estimates of outpatient antibiotic use for COVID-19.^6^ One potential explanation for the observed low rates of outpatient antibiotic prescription for COVID-19 is that the ubiquity of the COVID-19 pandemic resulted in public awareness of the lack of effective oral treatment options for early or mild disease. Additionally, the presence of widely available and highly sensitive SARS-COV-2 diagnostic testing may have allowed providers to take a “test and reassess” approach that has not been widely adopted for other viral illnesses. Thus, our findings highlight the potential role of accurate and rapid diagnostic tests to influence future antimicrobial prescription practices by reducing diagnostic uncertainty.^8,9^

The observed low antibiotic prescription rates could alternatively indicate a lower acuity of COVID-19 in our study population. However, the SARS-CoV-2–positive patients in our cohort had a 30-day admission rate of 2.9% within our hospital system only (Supplementary Table 2). This rate is comparable to the 1-month admission rate of 4.0%–9.0% described in a large outpatient cohort in France,^10^ suggesting that severity of COVID-19 disease in our patient population approximates that of other ambulatory settings.

This study had several limitations. We did not evaluate baseline data to demonstrate the rate of outpatient antibiotic prescriptions before the pandemic. We were also unable to determine the indications for patients’ ambulatory SARS-CoV-2 testing; thus, we could not compare antibiotic prescription rates in patients tested due to COVID-19–like symptoms and those who were asymptomatic. However, our study population reflects the real-world of SARS-CoV-2 testing because providers must interpret SARS-CoV-2 test results in asymptomatic or minimally symptomatic patients. Finally, we did not capture the diagnosis for which antibiotics were prescribed. Nevertheless, we were able to describe the clinical indications for all antibiotic prescriptions within 7 days of a positive SARS-CoV-2 test (Table 2).

In conclusion, we have described a low rate of outpatient antibiotic prescribing to patients within 7 days of a positive SARS-CoV2 test result over a 15-month period at a large public safety-net hospital system in the United States. Our results expand prior work illustrating low rates of outpatient antibiotic use during the COVID-19 pandemic and investigate the appropriateness of the antibiotic prescriptions among patients with COVID-19. The potential role for rapid diagnostics to improve outpatient antibiotic prescribing practices warrants further research.

## Supplementary material

For supplementary material accompanying this paper visit https://doi.org/10.1017/ash.2022.17. click here to view supplementary material
