# Supplementary material for: Low rates of antibiotic use among ambulatory patients with coronavirus disease 2019 (COVID-19)
Source: Antimicrob Steward Healthc Epidemiol. 2022 Apr 11;2(1):e62. doi: 10.1017/ash.2022.17 (PMC9119305; doi:10.1017/ash.2022.17)
Supplement: Supplementary file 1 [file S2732494X22000171sup001.docx]

**Supplemental Table 1.**Types of outpatient antibiotics prescribed, by COVID test result associated with prescription

| **Type of antibiotic,** *n (%)* | **Negative**, N = 1,326 | **Positive**, N = 55 |
| --- | --- | --- |
| Aminoglycosides | 3 (0.2%) | 0 (0%) |
| Cephalosporins | 148 (11%) | 2 (3.6%) |
| Fluoroquinolones | 143 (11%) | 2 (3.6%) |
| Macrolides | 84 (6.3%) | 18 (33%) |
| Penicillin | 509 (38%) | 15 (27%) |
| Tetracyclines | 3 (0.3%) | 0 (0%) |
| Clindamycin | 60 (4.5%) | 1 (1.8%) |
| Trimethoprim/sulfamethoxazole | 28**7** (22%) | 9 (16%) |
| Linezolid | 10 (0.8%) | 0 (0%) |
| Miscellaneous anti-infectives* | 176 (13%) | 12 (22%) |
| ***Notes:*** *Totals reflect the number of unique COVID testing instances; some patients received more than one type of antibiotic prescription with a single COVID testing occurrence.*  *All atovaquone, dapsone, and methamine hippurate prescriptions were excluded as prophylaxis. Trimethoprim/sulfamethoxazole (TMP/SMX) prescriptions among HIV+ patients with known CD4 cell counts <200 and all once-daily TMP/SMX prescriptions among HIV+ patients were also excluded as prophylaxis.*  **Miscellaneous anti-infectives included: metronidazole (n=96), nitrofurantoin (n=82), PO vancomycin (n=5), nitazoxanide (n=3), IV vancomycin (n=1), rifaximin (n=1)* | | |

**Supplemental Table 2.** Follow-up inpatient admission rates among COVID-positive outpatients who did vs. did not receive antibiotic prescriptions

|  | **All COVID+** | **No Abx** (n=1382) | **Abx** (n=55) | **p-value** |
| --- | --- | --- | --- | --- |
| 7-day inpatient admission proportion*^1^* (%) | 15/1,437 (1.0%) | 14/1,382 (1.0%) | 1/55 (1.8%) | NS |
| 30-day inpatient admission proportion (%) | 42/1,437 (2.9%) | 39/1,1382 (2.8%) | 3/55 (5.5%) | NS |
| Time to admission, days:  *Mean (SD)* | 13.43 (8.70) | 13.49 (8.75) | 12.67 (9.71) | NS |
| Length of admission, days: *^2^* *Mean (SD)* | 5.85 (9.33) | 4.68 (4.27) | 20.67 (32.39) | 0.003 |
| Admissions with ICU stay (%) | 11/42 (26.2%) | 10/39 (25.6%) | 1/3 (33.3%) | NS |
| Duration of ICU stay, days: *Mean (SD)* | 9.18 (16.51) | 4.30 (3.40) | 58.0 (NA) | -- |
| *^1^* Inpatient admissions did not include admissions for elective orthopedic surgery as these were not coded as inpatient admissions in the GHS Epic database.  *^2^*One patient with an admission date but no discharge date was excluded from this calculation. | | | | |
